# Metabolic and Anthropometric Effects of a Randomized Freely Chosen Exercise Prescription Program vs a Video-Based Training Program in Patients With Childhood Obesity: A Randomized Clinical Trial

**DOI:** 10.7759/cureus.81287

**Published:** 2025-03-27

**Authors:** Karen Pedraza-Escudero, Nayely Garibay-Nieto, Eréndira Villanueva-Ortega, Carlos Omar López-López, Rebeca Galindo-Díaz, Adán Germán Gallardo-Rodríguez, Gloria Eugenia Queipo-García, Alejandra Ruíz-Barranco, María José Garcés-Hernández, Mireya León-Hernández, Estibalitz Laresgoiti-Servitje

**Affiliations:** 1 Pediatric Obesity Clinic at Child Wellness Unit, Hospital General de México “Dr. Eduardo Liceaga", Mexico City, MEX; 2 Pediatric Obesity Clinic at Child Wellness Unit, Hospital General de México “Dr. Eduardo Liceaga”, Mexico City, MEX; 3 Applied Research and Technology Institute (InIAT), Universidad Iberoamericana, Mexico City, MEX; 4 Center for Continuing Education and Advanced Sports Studies, Universidad Nacional Autónoma de México, Mexico City, MEX; 5 Hematology Research Department, Hospital General de México “Dr. Eduardo Liceaga”, Mexico City, MEX; 6 Medical Genetics Service, NanoLab Next Generation Diagnostics, Mexico City, MEX; 7 Research Unit, Hospital General de México “Dr. Eduardo Liceaga”, Mexico City, MEX; 8 Clinical Sciences, School of Medicine and Health Sciences, Tecnológico de Monterrey, Mexico City, MEX

**Keywords:** childhood obesity, clinical trial, exercise, lifestyle changes, physical activity

## Abstract

Objectives

When dealing with children and adolescents living with obesity, it is vital to be aware that exercise provides benefits in the prevention and control of non-communicable diseases and general well-being. The growing prevalence of childhood obesity makes it necessary to develop strategies aimed at controlling the barriers that limit the performance of exercise, which is why we designed a plan of exercise prescription through videos that could be accessible, free, and designed for exercise at home, as a complement to a multidisciplinary intervention program for lifestyle change. This study aimed to compare the effects of a video-based exercise prescription program (EPV) versus free-choice exercise (FCE) on anthropometric and metabolic indicators.

Methods

We conducted an open-label, randomized, controlled clinical trial. Patients aged eight to 16 years with obesity from the Child Unit of the General Hospital of Mexico were included. Forty-two participants finished the follow-up; 20 were boys, and 22 were girls. All patients were included in a multi-component program of healthy lifestyle changes and randomized to receive EPV (n=22) or FCE (n=20) for six months.

Results

The primary outcomes in both groups were a decrease in body mass index (BMI) (p < 0.001), a reduction in body fat mass (p < 0.001), and an increase in lean body mass (p = 0.003). Other outcomes observed were: in EPV, there was a decrease in low density lipid (LDL) (p=0.04); alanine aminotransferase (ALT) (p=0.002), aspartate aminotransferase (AST) (p=0.001) and uric acid (p=0.003) and an increase in high density lipid (HDL) (p=0. 002), while in FCE there was a decrease in Homeostatic Model Assessment for Insulin Resistance (HOMA-IR) (p=0.006), insulin (p=0.006), LDL (p=0.02), ALT (p=0.002), AST (p=0.004) and gamma-glutamyl transferase (GGT) (p=0.025).

Conclusion

Both EPV and FCE exercise prescription programs, as part of a multidisciplinary intervention for childhood obesity, had favorable effects on body composition and metabolic parameters.

## Introduction

Childhood obesity is one of the most important global public health problems. The global prevalence of obesity by 2022 in school children and adolescents was estimated at 159.3 million, which increased from 1990 to 2022, more than doubled globally [1]. The prevalence of obesity in Mexico in 2022 was estimated at 18.1% in school children aged five to 11 years. The prevalence of obesity in adolescents aged 12 to 19 years in 2022 was 17.2% [2]. In Mexico, there has also been a growing prevalence of childhood obesity. Between 2006 and 2022, it increased by 24%. In adolescents, the prevalence of obesity increased by 50% from 2006 to 2022 [2].

Obesity is a complex disease influenced by genetic, socioecological, political, economic, and environmental factors [3]. The severity of the disease is associated with a large number of comorbidities, such as metabolic syndrome, hypertension, dyslipidemia, insulin resistance, type 2 diabetes mellitus, steatotic liver disease, as well as musculoskeletal and psychological disturbances [4-6]. Therefore, it is essential to carry out appropriate prevention and intervention strategies. The health care model should be multidisciplinary, including educational workshops, modification of healthy eating habits and physical activity, and care by specialists who correctly assess the comorbidities and needs of patients [7,8].

Interventions are widely established in nutritional management. However, adequate guidance on physical activity and exercise prescription in children and adolescents living with obesity is limited due to a lack of knowledge of an adequate prescription that provides the required therapeutic effect, which is also affected by the lifestyles of the population [3].

The World Health Organization (WHO) Guidelines on Physical Activity and Sedentary Behaviour for Health recommend that children and adolescents aged five to 17 years engage in at least 60 minutes of moderate to vigorous physical activity daily [9]. Accordingly, global estimates show that physical activity trends are insufficient; 81% of school children and adolescents aged 11-17 did not engage in sufficient physical activity in 2016 [10].

According to the WHO recommendation, in Mexico, the National Health and Nutrition Survey 2022 estimated that in the population of children aged 10-14 years, 68.3% have insufficient physical activity, and up to 82.2% spend more than two hours a day in front of screens. As for adolescents aged 15-19, 42.6% have insufficient physical activity, and 90.8% spend more than two hours a day in front of screens [11]. 

Physical activity and exercise are imperative to improve or maintain physical fitness, defined as the ability to perform physical activity without experiencing excessive fatigue or pain [12]. Physical inactivity can negatively affect health-related components of physical fitness, such as decreased aerobic capacity, muscle strength and tolerance, flexibility, and balance [12]. Therefore, it is crucial to detect the different physical barriers to movement and provide appropriate rehabilitation intervention, avoiding the incidence of risks associated with physical activity, such as musculoskeletal injuries or exacerbation of underlying problems [12,13].

It is essential to be clear about the benefits of exercise in children and adolescents living with obesity and its importance as a cornerstone in the prevention and control of non-communicable diseases, cardiovascular disease, and musculoskeletal health, as well as the impact on somatic, motor, social, emotional and intellectual development [14,15].

To obtain the benefits of exercise in children and adolescents, an accurate exercise prescription must be made following the dose-response relationship between physical activity and health effects, whereby the training program must be based on the principles of physical training: Individualization, Specificity, Variability, Overload, Progression. Finally, it should be considered that if there is no adequate stimulus, there will be a reversibility of the adaptations already obtained [16]. The training program should establish and describe the following variables corresponding to the components of the training: the Frequency and Intensity at which the exercise will be performed, the Time, and the Type of exercise to be performed (FITT). In children and adolescents, the following should be added to complement the exercise prescription: Progression, Precautions, and Entertainment or fun (PPE) when performing the exercise. The latter is fundamental for creating positive and pleasurable exercise experiences in the early stages of life. It should consider age, preferences, individual objectives, the availability of physical facilities for the exercise, and the characteristics of the environment [17,18].

Before starting an exercise program, it is essential to carry out a detailed medical evaluation, including clinical history, physical and musculoskeletal examination, biomechanical evaluation, evaluation of the level of physical activity and pre-existing physical condition, cardiovascular risk screening, nutritional evaluation, and evaluation of motivation to change [13,18].

Studies conducted in children and adolescents generally show beneficial effects. Some studies have delved into the effect of exercise on body composition parameters and cardiometabolic risk, among others, before different types of exercise and dose/response to it [19-21]. However, the evidence regarding exercise prescription in children and adolescents living with obesity is still insufficient, so there is a need for further studies on the threshold effect of exercise and cardiometabolic health produced by different combinations of intensity, duration, frequency, and type of exercise [22]. 

Current evidence highlights the influence of the social environment on people's physical activity levels. The school environment and the promotion of physical activity through the health system (in hospitals, clinics, and health centers) have been shown to significantly impact the behavior and health of children and adolescents, influencing the levels of physical activity they engage in [23].

Given the above background, the growing demand for care in the childhood obesity clinic of the General Hospital of Mexico, and the sociocultural characteristics of our patients, we are faced with the challenge of creating a strategy to break down the barriers to exercise in our Mexican population, such as the lack of economic resources to attend extracurricular classes, complex family dynamics, the absence of support and supervision in the activities, inadequate or problematic to access safe spaces for exercise, as well as the increased insecurity on public roads and the lack of knowledge of the proper performance of exercise [11,19,20,23].

In accordance with the needs of our population, we designed a strategy for prescribing exercise through videos, which could be accessible, free of charge, and designed to be done at home, in a small space, and with simple elements for carrying out the movements. This is in addition to the multidisciplinary intervention program for changes in a healthy lifestyle that we carry out in our Child Wellness Unit. This study aimed to compare the effects of an exercise prescription program, through videos vs.* *free-choice exercise, on anthropometric and metabolic indicators, following the principles of physical training.

## Materials and methods

Our study is a randomized, open-label, controlled clinical trial, with two parallel arms of video-based exercise prescription compared to free-choice exercise. The study was conducted following the Declaration of Helsinki and approved by the Institutional Ethics Committee of Hospital General de México, "Dr. Eduardo Liceaga" protocol code DI/17/311/03/028, date of approval: April 25th, 2017. Three hundred and fifty patients from the Child Wellness Unit at the Hospital General de México "Dr. Eduardo Liceaga" were assessed for eligibility from October 2017 to February 2020. This protocol and its database are registered on the Clinical Trials page with the number NCT03552367. The clinical trial protocol included as Supplementary Information is the version submitted to and approved by the Ethics Committee before the trial began.

**Figure 1:**
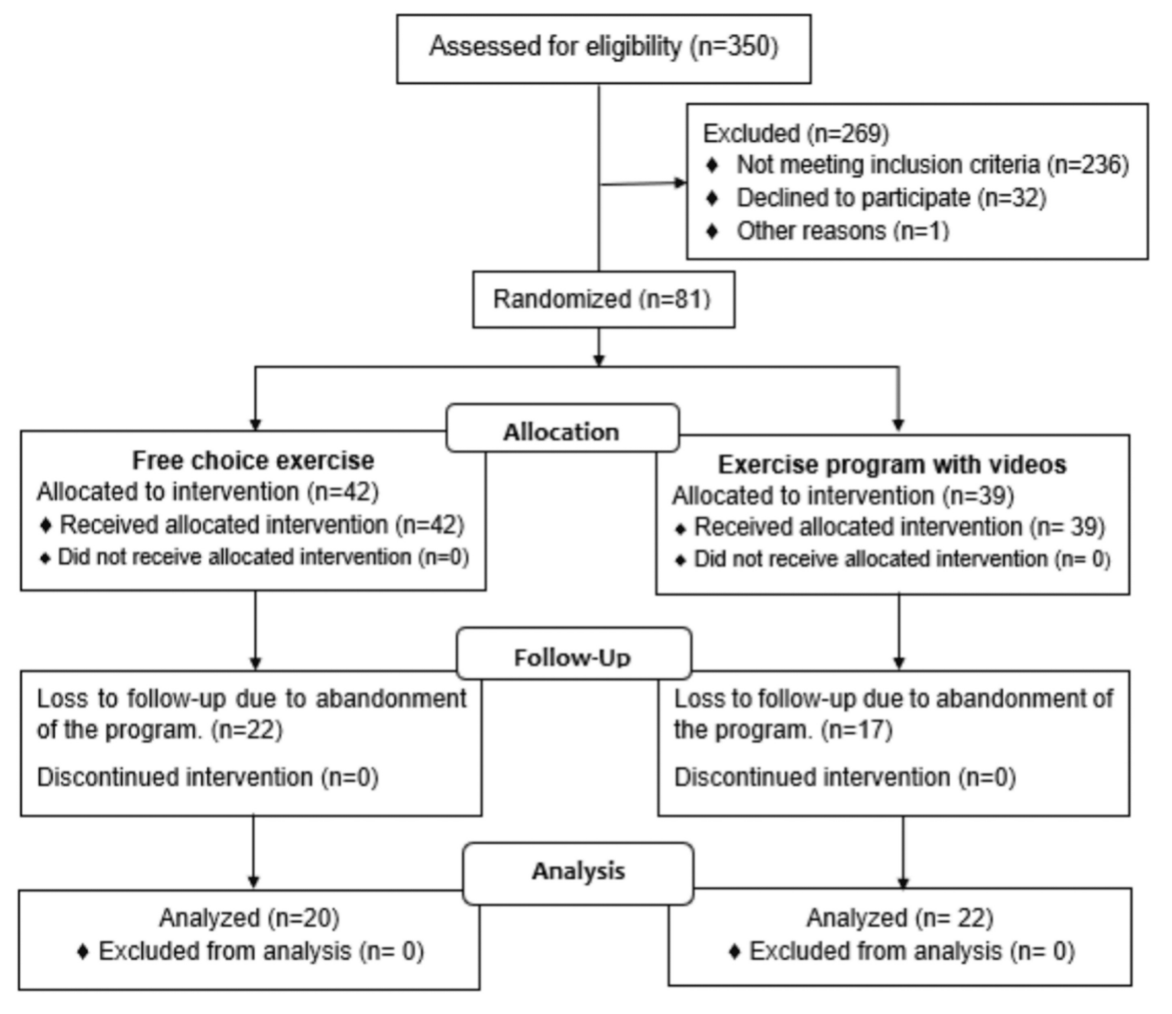
Participant Flow Diagram

Eighty-one patients who met the inclusion criteria were recruited. Participants ranged from eight to 16 years. Forty-two participants finished the follow-up; 20 were boys, and 22 were girls. All of them had a diagnosis of class 1 obesity, determined by BMI ≥95th percentile and < 120% of 95th percentile according to the criteria established by the Centers for Disease Control and Prevention [24]. We excluded patients with obesity of genetic origin or with a previously known endocrinological disease, primary dyslipidemia, patients on pharmacological treatment that potentially modified lipid or carbohydrate metabolism, presence of arterial hypertension (≥ 95th percentile for sex, age, and height), infectious and systemic diseases at the time of the study, history of acute or prolonged immobilization, orthopedic or neurological limitations that limited the performance of the tests, and history of any previous intervention. In this study, we obtained written informed consent and assent from all subjects and their parents/legal guardians, with the signatures of two witnesses. Every participant was included in a multi-component program of healthy lifestyle changes and randomized to one of the two intervention groups, EPV or FCE, for six months (Figure 1).

Multi-component intervention program for healthy lifestyle changes

During the trial, the participants of both programs received the multi-component program that is usually carried out at the Child Wellness Unit, which consists of one first assessment and five follow-up evaluations by the multidisciplinary team made up of Pediatricians specialized in Child and Adolescent Obesity, Pediatric Endocrinologists, Nutritionists, Psychologists, Sports Doctors, and Physical Rehabilitators.

After the initial consultation, we conducted five follow-up consultations, including an exercise session, a psychoeducational workshop, and a medical and nutritional consultation. Regarding the joint exercise session for patients and their parents, we seek to encourage them to reduce their sedentary lifestyle, increase their activity levels, and teach them how to exercise correctly, either through recreational activities or free-choice exercise. These sessions are carried out and supervised by a medical expert who performs exercise prescriptions to evaluate the correct execution of the exercise. Afterward, a psychoeducational workshop is given, where nutrition, psychological, physical activity, sedentary lifestyle, and complications associated with overweight and obesity are discussed. Subsequently, the patient receives medical consultation and nutritional follow-up, where anthropometric evaluations are made, goals are established, feedback is given on changes to a healthy lifestyle, and treatment is prescribed for each patient to maintain an adequate growth rate and achieve a healthier weight-height ratio. If required, assessment and follow-up by the psychology team are performed.

Anthropometric measurements and body composition

A pediatrician or pediatric endocrinologist first evaluated participants. The clinical variables assessed were age, sex, weight, standing height, BMI, waist circumference, stage of pubertal development, vital signs, and body composition, which were assessed during the six visits of the intervention.

Body weight and body composition measurements were made by electrical bioimpedance (Body Composition Analyzer Model IOI 353 to 0.1 kg) Jawon Medical Co, Gyeonggi-do, Republic of Korea) after an 8-12 hour fast, with the patient barefoot and wearing light clothing. Height was measured employing a stadiometer (SECA mobile model 217, SECA GmbH & Co., Hamburg, Germany) with the patient barefoot; the measuring plate was placed on the vertex when the head was in the Frankfurt plane position. Weight and height were measured to the nearest 0.1 in kg and cm. The waist circumference was measured at the end of an exhalation with a nonelastic flexible tape (CESCORF model 019213315337, Brazil) in the standing position at the level of the midpoint between the lower costal border and the iliac crest. Pediatricians and pediatric endocrinologists performed physical examinations of pubertal developmental status, according to the Tanner scale [25,26]. Blood pressure was measured in duplicate while seated over the right brachial artery using a manual sphygmomanometer and specially-sized pressure cuffs according to the patient's age [27].

Measurement of biochemical parameters

We took a peripheral venous blood sample with fasting of 12 hrs; enzymatic methods were performed for the determination of glucose, total cholesterol, high-density lipoprotein (HDL) cholesterol, low-density lipoprotein (LDL) cholesterol, triglycerides, uric acid, alanine aminotransferase (ALT) and aspartate aminotransferase (AST), gamma-glutamyltranspeptidase (GGT), oral glucose tolerance curve and insulin curve. Insulin was determined by chemiluminescence analysis.

We performed an oral glucose tolerance curve by calculating 1.75 g of glucose per kilogram in subjects under 43 kg. In comparison, subjects with a higher body weight were administered 75 g in a single dose. We took samples at 0, 30, 60, 90, and 120 minutes to measure glucose and insulin concentration.

We calculated insulin resistance using the indices of the homeostatic model for assessing insulin resistance (HOMA-IR), using the following equation: (fasting insulin mU/L x fasting glucose mg/dl/405) [28]. We performed all measurements at the beginning and the end of the intervention.

Nutritional evaluation

A nutritionist with expertise in childhood obesity performed a complete nutritional assessment consisting of an eating habits questionnaire and a 24-hour recall of ordinary days and weekends. Each patient was assigned a structured dietary plan based on the recommendations for healthy eating proposed for Mexico in the Draft Mexican Official Standard NOM-043-SSA2-2012 [29] based on age, gender, pubertal developmental stage, and degree of physical activity. The energy requirement was calculated using the Schofield equation with weight and height [30]. In order to ensure adequate energy intake, an isocaloric and isoproteic food plan was made, with the following macronutrient distribution: proteins 20%, lipids 25%, and carbohydrates 55% [31].

The nutritional intervention was carried out at each follow-up visit; at each session, eating behavior and adherence to the dietary plan were evaluated through a food record diary, adjusting caloric intake according to the degree of physical activity performed by each patient. In these sessions, feasible objectives were planned according to the patient and his family, giving feedback focused on changes in healthy eating habits, the nutritional counseling aimed at modifying unhealthy eating behavior by increasing the consumption of fresh fruits, vegetables, whole grains, legumes, fish, eggs and decreasing simple carbohydrates, as well as reducing processed and industrialized foods.

Sports medicine and rehabilitation assessment

A biomechanical evaluation was performed, which consisted of an assessment of posture, gait, arcs of joint mobility, inspection with a somatoscope in a frontal and lateral manner to evaluate body symmetry and proportions to identify any changes that could contraindicate exercise and/or the need to use orthoses to avoid musculoskeletal injuries or aggravate underlying problems. Physical condition was also evaluated, including flexibility through trunk anteroflexion, sitting without shoes, and resting the soles of the feet against the flexometer. The most distant point reached with the fingertips, capable of being maintained for at least two seconds out of two attempts, was taken as the value of the test. Upper limb strength was measured by manual dynamometry, and lower limb strength was evaluated by horizontal jump [12].

Subsequently, a sports physician from the Sports Medicine Unit of the National Autonomous University of Mexico (UNAM) evaluated the patient's physical condition through the Bruce treadmill protocol to determine the presence of cardiovascular risk during exercise and recovery and to determine the maximum heart rate to prescribe and monitor the intensity of the exercise. During the test, electrocardiographic monitoring was carried out, as well as monitoring of heart rate, blood pressure, and the participants' feelings of fatigue [12].

Freely-chosen exercise

Each participant randomized to the FCE chose the type of exercise to be performed according to their preferences and accessibility; our patients chose those listed below: swimming, dancing, athletics, soccer, basketball, and exercise videos from the Internet. The participants who were included in this intervention group, to favor adaptation to exercise and improve physical condition, were instructed to exercise at a frequency of at least three times per week, with a minimum duration of 40 minutes, at an intensity of 55-60% of their maximum heart rate or 3-4 metabolic equivalents (METs). At each visit, the progression of the exercise was indicated, according to the tolerance and evolution of each participant, until reaching the execution of five to six times per week with a duration of 1 hour and 30 minutes of exercise, at an intensity of 65-75% of the maximum heart rate or 5-6.9 METs. During the intervention, each participant was provided with a heart rate monitor (Polar Ft7, Polar Electro, Kempele, Finland) to dose and objectively control the intensity of the exercise and record the adherence to the exercise program in terms of frequency, intensity, and duration of the exercise session.

Exercise program through videos

The EPV participants were asked to participate in a program designed especially for this research project, which included video-based exercise sessions focusing on child or adolescent patients; this program involved the collaboration of pediatricians, pediatric obesologists, rehabilitation specialists, and sports medicine specialists. The videos were distributed on compact discs or USB memory sticks. The program required participants to initially exercise three days a week for 40 minutes at an intensity of 55-60% of their maximum heart rate or 3-4 METs; a monthly progression was made according to the tolerance and evolution of each participant until reaching the goal of 5-6 times a week with a duration of 1 hour 30 minutes of exercise, at an intensity of 65-75% of the maximum heart rate or 5-6.9 METs. Likewise, each participant was provided with a heart rate monitor (Polar Ft7, Polar Electro, Kempele, Finland) to dose and objectively control the exercise's intensity and record the exercise program's adherence in frequency, intensity, and duration of the exercise session.

The training sessions were composed as follows: 1) Theoretical and technical preparation, where it was explained what the training session would consist of and the elements to be used, such as appropriate clothing and footwear for exercise, mat, jump rope, or ball, with emphasis on having simple water available for hydration, 2) Warm-up, in which low-intensity muscular movements and cardiovascular activities were performed, including coordination exercises, 3) Core phase, which consisted of a) aerobic exercise in the form of circuits with established time so that each participant could regulate the intensity indicated concerning their maximum heart rate, b) strength exercises, which were performed mainly directed to upper limbs, trunk, and lower limbs; the exercises were performed with their body weight and progressively using elements such as balls and resistance bands. 4) Cool-down, including cardiovascular and low-muscle tolerance activities, combining coordination and balance exercises, 5) Stretching and passive static exercises involving all muscle groups, ended the training session. The following figures provide a detailed overview of the training program carried out through videos, describing each of the microcycles, the progression in frequency, intensity, time, and variety of each training session, and the respective breaks and times for hydration (Tables 1-5).

**Table 1:** First microcycle Duration of training session: 40 minutes, Intensity: 55-60% HRmax (3-4 METs). HRmax: maximum heart rate; Min: minutes; MET: Metabolic equivalents.

Day 1	Day 2	Day 3	Day 4	Day 5	Day 6	Day 7
Preparation: Theoretical 2 min. Technical 2 min.		Preparation: Theoretical 2 min. Technical 2 min.		Preparation: Theoretical 2 min. Technical 2 min.		
Warm-up 8 min.		Warm-up 8 min.		Warm-up 8 min.		
Active rest & hydration 1 min.		Active rest & hydration 1 min.		Active rest & hydration 1 min.		
Conditioning: Aerobic exercise	Active rest (physical activity)	Conditioning: Aerobic exercise	Active rest (physical activity)	Conditioning: Aerobic exercise	Active rest (physical activity)	Active rest (physical activity)
Intensity: 55-60% HRmax		Intensity: 55-60% HRmax		Intensity: 55-60% HRmax		
Circuit x 4 min.		Circuit x 4 min.		Circuit x 4 min.		
Active rest & hydration 2 min.		Active rest & hydration 2 min.		Active rest & hydration 2 min.		
Circuit x 4 min.		Circuit x 4 min.		Circuit x 4 min.		
Active rest & hydration 2 min.		Active rest & hydration 2 min.		Active rest & hydration 2 min.		
Cool-down 5 min.		Cool-down 5 min.		Cool-down 5 min.		
Stretching 10 min.		Stretching 10 min.		Stretching 10 min.		
Hydration		Hydration		Hydration		

**Table 2:** Second microcycle Duration of training session: 50 minutes, Intensity: 60-65% HRmax (4-5 METs). HRmax: maximum heart rate; Min: minutes; MET: Metabolic equivalents.

Day 1	Day 2	Day 3	Day 4	Day 5	Day 6	Day 7
Preparation: Theoretical 2 min. Technical 2 min.		Preparation: Theoretical 2 min. Technical 2 min.		Preparation: Theoretical 2 min. Technical 2 min.		Preparation: Theoretical 2 min. Technical 2 min.
Warm-up 8 min.		Warm-up 8 min.		Warm-up 8 min.		Warm-up 8 min.
Active rest & hydration 1 min.		Active rest & hydration 1 min.		Active rest & hydration 1 min.		Active rest & hydration 1 min.
Conditioning: Aerobic exercise	Active rest (physical activity)	Conditioning: Aerobic exercise	Active rest (physical activity)	Conditioning: Aerobic exercise	Active rest (physical activity)	Conditioning: Aerobic exercise
Intensity: 60-65% HRmax		Intensity: 60-65% HRmax		Intensity: 60-65% HRmax		Intensity: 60-65% HRmax
Circuit x 4 min.		Circuit x 4 min.		Circuit x 4 min.		Circuit x 4 min.
Active rest & hydration 2 min.		Active rest & hydration 2 min.		Active rest & hydration 2 min.		Active rest & hydration 2 min.
Circuit x 4 min.		Circuit x 4 min.		Circuit x 4 min.		Circuit x 4 min.
Active rest & hydration 2 min.		Active rest & hydration 2 min.		Active rest & hydration 2 min.		Active rest & hydration 2 min.
Circuit x 4 min.		Circuit x 4 min.		Circuit x 4 min.		Circuit x 4 min.
Active rest & hydration 3 min.		Active rest & hydration 3 min.		Active rest & hydration 3 min.		Active rest & hydration 3 min.
Resistance exercises General muscle exercise		Resistance exercises General muscle exercise		Resistance exercises General muscle exercise		Resistance exercises General muscle exercise
Upper limb strength exercises: 3 exercises 8 repetitions 3 sets		Upper limb strength exercises: 3 exercises 8 repetitions 3 sets		Upper limb strength exercises: 3 exercises 8 repetitions 3 sets		Upper limb strength exercises: 3 exercises 8 repetitions 3 sets
Back & abdominal strength exercises: 3 exercises 8 repetitions 3 sets		Back & abdominal strength exercises: 3 exercises 8 repetitions 3 sets		Back & abdominal strength exercises: 3 exercises 8 repetitions 3 sets		Back & abdominal strength exercises: 3 exercises 8 repetitions 3 sets
Lower limb strength exercises: 3 exercises 8 repetitions 3 sets		Lower limb strength exercises: 3 exercises 8 repetitions 3 sets		Lower limb strength exercises: 3 exercises 8 repetitions 3 sets		Lower limb strength exercises: 3 exercises 8 repetitions 3 sets
Hydration		Hydration		Hydration		Hydration
Cool-down 5 min.		Cool-down 5 min.		Cool-down 5 min.		Cool-down 5 min.
Stretching 12 min.		Stretching 12 min.		Stretching 12 min.		Stretching 12 min.
Hydration		Hydration		Hydration		Hydration

**Table 3:** Third microcycle Duration of training session: 65 minutes, Intensity: 65-70% HRmax (5-6 METs). HRmax: maximum heart rate; Min: minutes; MET: Metabolic equivalents.

Day 1	Day 2	Day 3	Day 4	Day 5	Day 6	Day 7
Preparation Theoretical 2 min. Technical 4 min.		Preparation Theoretical 2 min. Technical 4 min.		Preparation Theoretical 2 min. Technical 4 min.		Preparation Theoretical 2 min. Technical 4 min.
Warm-up 10 min.		Warm-up 10 min.		Warm-up 10 min.		Warm-up 10 min.
Active rest & hydration 2 min.		Active rest & hydration 2 min.		Active rest & hydration 2 min.		Active rest & hydration 2 min.
Conditioning: Aerobic exercise	Active rest (physical activity)	Conditioning: Aerobic exercise	Active rest (physical activity)	Conditioning: Aerobic exercise	Active rest (physical activity)	Conditioning: Aerobic exercise
Intensity: 65-70% HRmax		Intensity: 65-70% HRmax		Intensity: 65-70% HRmax		Intensity: 65-70% HRmax
Circuit x 8 min.		Circuit x 8 min.		Circuit x 8 min.		Circuit x 8 min.
Active rest & hydration 2 min.		Active rest & hydration 2 min.		Active rest & hydration 2 min.		Active rest & hydration 2 min.
Circuit x 8 min.		Circuit x 8 min.		Circuit x 8 min.		Circuit x 8 min.
Active rest & hydration 2 min.		Active rest & hydration 2 min.		Active rest & hydration 2 min.		Active rest & hydration 2 min.
Jump rope circuit x 5 min.		Jump rope circuit x 5 min.		Jump rope circuit x 5 min.		Jump rope circuit x 5 min.
Active rest & hydration 3 min.		Active rest & hydration 3 min.		Active rest & hydration 3 min.		Active rest & hydration 3 min.
Resistance exercises General muscle exercise		Resistance exercises General muscle exercise		Resistance exercises General muscle exercise		Resistance exercises General muscle exercise
Upper limb strength exercises: 3 exercises 10 repetitions 3 sets		Upper limb strength exercises: 3 exercises 10 repetitions 3 sets		Upper limb strength exercises: 3 exercises 10 repetitions 3 sets		Upper limb strength exercises: 3 exercises 10 repetitions 3 sets
Back & abdominal strength exercises: 3 exercises 10 repetitions 3 sets		Back & abdominal strength exercises: 3 exercises 10 repetitions 3 sets		Back & abdominal strength exercises: 3 exercises 10 repetitions 3 sets		Back & abdominal strength exercises: 3 exercises 10 repetitions 3 sets
Lower limb strength exercises: 3 exercises 10 repetitions 3 sets		Lower limb strength exercises: 3 exercises 10 repetitions 3 sets		Lower limb strength exercises: 3 exercises 10 repetitions 3 sets		Lower limb strength exercises: 3 exercises 10 repetitions 3 sets
Hydration		Hydration		Hydration		Hydration
Cool-down 5 min.		Cool-down 5 min.		Cool-down 5 min.		Cool-down 5 min.
Stretching 15 min.		Stretching 15 min.		Stretching 15 min.		Stretching 15 min.
Hydration		Hydration		Hydration		Hydration

**Table 4:** Fourth microcycle Duration of training session: 80 minutes, Intensity: 65-70% HRmax (5-6 METs). HRmax: maximum heart rate; Min: minutes; MET: Metabolic equivalents.

Day 1	Day 2	Day 3	Day 4	Day 5	Day 6	Day 7
Preparation: Theoretical 2 min. Technical 4 min.	Preparation: Theoretical 2 min. Technical 4 min.	Preparation: Theoretical 2 min. Technical 4 min.	Preparation: Theoretical 2 min. Technical 4 min.	Preparation: Theoretical 2 min. Technical 4 min.		
Warm-up 10 min.	Warm-up 10 min.	Warm-up 10 min.	Warm-up 10 min.	Warm-up 10 min.		
Active rest & hydration 2 min.	Active rest & hydration 2 min.	Active rest & hydration 2 min.	Active rest & hydration 2 min.	Active rest & hydration 2 min.		
Conditioning: Aerobic exercise	Conditioning: Aerobic exercise	Conditioning: Aerobic exercise	Conditioning: Aerobic exercise	Conditioning: Aerobic exercise	Active rest (physical activity)	Active rest (physical activity)
Intensity: 65-70% HRmax	Intensity: 65-70% HRmax	Intensity: 65-70% HRmax	Intensity: 65-70% HRmax	Intensity: 65-70% HRmax		
Circuit x 10 min.	Circuit x 10 min.	Circuit x 10 min.	Circuit x 10 min.	Circuit x 10 min.		
Active rest & hydration 2 min.	Active rest & hydration 2 min.	Active rest & hydration 2 min.	Active rest & hydration 2 min.	Active rest & hydration 2 min.		
Circuit x 10 min.	Circuit x 10 min.	Circuit x 10 min.	Circuit x 10 min.	Circuit x 10 min.		
Active rest & hydration 2 min.	Active rest & hydration 2 min.	Active rest & hydration 2 min.	Active rest & hydration 2 min.	Active rest & hydration 2 min.		
Jump rope circuit x 6 min.	Jump rope circuit x 6 min.	Jump rope circuit x 6 min.	Jump rope circuit x 6 min.	Jump rope circuit x 6 min.		
Active rest & hydration 3 min.	Active rest & hydration 3 min.	Active rest & hydration 3 min.	Active rest & hydration 3 min.	Active rest & hydration 3 min.		
Resistance exercises	Resistance exercises	Resistance exercises	Resistance exercises	Resistance exercises		
Muscles exercise of the lower body: 6 exercises 10 repetitions 3 sets	Exercise of the core muscles: 6 exercises 10 repetitions 3 sets	Muscles exercise of upper body: 6 exercises 10 repetitions 3 sets	Muscles exercise of the lower body: 6 exercises 10 repetitions 3 sets	Exercise of the core muscles: 6 exercises 10 repetitions 3 sets		
Hydration	Hydration	Hydration	Hydration	Hydration		
Cool-down 5 min.	Cool-down 5 min.	Cool-down 5 min.	Cool-down 5 min.	Cool-down 5 min.		
Stretching 15 min.	Stretching 15 min.	Stretching 15 min.	Stretching 15 min.	Stretching 15 min.		
Hydration	Hydration	Hydration	Hydration	Hydration		

**Table 5:** Fifth Microcycle Duration of training session: 90 minutes, Intensity: 65-75% HRmax (5-6.9 METs). HRmax: maximum heart rate, Min: minutes, MET: Metabolic equivalents.

Day 1	Day 2	Day 3	Day 4	Day 5	Day 6	Day 7
Preparation: Theoretical 2 min. Technical 4 min.	Preparation: Theoretical 2 min. Technical 4 min.	Preparation: Theoretical 2 min. Technical 4 min.	Preparation: Theoretical 2 min. Technical 4 min.	Preparation: Theoretical 2 min. Technical 4 min.	Preparation: Theoretical 2 min. Technical 4 min.	
Warm-up 10 min.	Warm-up 10 min.	Warm-up 10 min.	Warm-up 10 min.	Warm-up 10 min.	Warm-up 10 min.	
Active rest & hydration 2 min.	Active rest & hydration 2 min.	Active rest & hydration 2 min.	Active rest & hydration 2 min.	Active rest & hydration 2 min.	Active rest & hydration 2 min.	
Conditioning: Aerobic exercise	Conditioning: Aerobic exercise	Conditioning: Aerobic exercise	Conditioning: Aerobic exercise	Conditioning: Aerobic exercise	Conditioning: Aerobic exercise	Active rest (physical activity
Intensity: 65-75% HRmax	Intensity: 65-75% HRmax	Intensity: 65-75% HRmax	Intensity: 65-75% HRmax	Intensity: 65-75% HRmax	Intensity: 65-75% HRmax	
Circuit x 12 min.	Circuit x 12 min.	Circuit x 12 min.	Circuit x 12 min.	Circuit x 12 min.	Circuit x 12 min.	
Active rest & hydration 2 min.	Active rest & hydration 2 min.	Active rest & hydration 2 min.	Active rest & hydration 2 min.	Active rest & hydration 2 min.	Active rest & hydration 2 min.	
Circuit x 12 min.	Circuit x 12 min.	Circuit x 12 min.	Circuit x 12 min.	Circuit x 12 min.	Circuit x 12 min.	
Active rest & hydration 2 min.	Active rest & hydration 2 min.	Active rest & hydration 2 min.	Active rest & hydration 2 min.	Active rest & hydration 2 min.	Active rest & hydration 2 min.	
Jump rope circuit x 6 min.	Jump rope circuit x 6 min.	Jump rope circuit x 6 min.	Jump rope circuit x 6 min.	Jump rope circuit x 6 min.	Jump rope circuit x 6 min.	
Active rest & hydration 3 min.	Active rest & hydration 3 min.	Active rest & hydration 3 min.	Active rest & hydration 3 min.	Active rest & hydration 3 min.	Active rest & hydration 3 min.	
Resistance exercises	Resistance exercises	Resistance exercises	Resistance exercises	Resistance exercises	Resistance exercises	
Muscles exercise of the lower body: 6 exercises 10 repetitions 3 sets	Exercise of the core muscles: 6 exercises 10 repetitions 3 sets	Muscles exercise of upper body: 6 exercises 10 repetitions 3 sets	Muscles exercise of the lower body: 6 exercises 10 repetitions 3 sets	Exercise of the core muscles: 6 exercises 10 repetitions 3 sets	Muscles exercise of upper body: 6 exercises 10 repetitions 3 sets	
Hydration	Hydration	Hydration	Hydration	Hydration	Hydration	
Cool-down 5 min.	Cool-down 5 min.	Cool-down 5 min.	Cool-down 5 min.	Cool-down 5 min.	Cool-down 5 min.	
Stretching 15 min.	Stretching 15 min.	Stretching 15 min.	Stretching 15 min.	Stretching 15 min.	Stretching 15 min.	
Hydration	Hydration	Hydration	Hydration	Hydration	Hydration	

Randomization and blinding

A 1:1 randomization process was performed, without stratification and permuted blocks, using a random number table with the Excel program version 2016 for Mac. The participants were randomly divided into two groups, EPV and FCE. At baseline and after the intervention period, all assessments were performed by the specialists, who were unaware of the groups throughout the study.

Statistical analysis

We performed descriptive statistics and evaluated normality with a Shapiro-Wilk test, explored the main variables with box and whisker plots to detect outliers, and performed Levene's test to assess the homogeneity of variances. No participants were excluded from the analysis. We compared the two groups' baseline characteristics, employing a two-tailed Student's t-test for independent samples for quantitative variables with normal distribution and the Mann-Whitney U test for those with non-normal distribution. For categorical variables, we used the Chi-square test. The three liver enzyme variables (AST, ALT, GGT) were Log10 transformed for the analyses of variance due to violation of the normality assumption and positive asymmetry.

In order to demonstrate statistically significant differences between the intervention groups, we initially performed analyses of variance (ANOVA) to assess differences between and within groups (time and treatment) and time x group interactions. Secondarily, repeated measures one-way analyses of covariance (ANCOVA) were executed for the anthropometric variables, adjusting for the value of the initial evaluation; analyses of covariance (ANCOVA) were also used for metabolic variables, controlling for the value of the first assessment.

To evaluate the difference between the beginning and the end of the intervention of anthropometric and metabolic variables of each group, we used Student's t-test for related samples for variables with normal distribution and for Log10 transformed variables. All analyses were performed with SPSS Statistics software, version 29 (IBM Corp., Armonk, NY) [32]. All statistical tests were two-tailed, and statistical significance was determined by a p-value <0.05.

## Results

Demographic, anthropometric, and metabolic characteristics of patients.

Eighty-one pediatric patients with class 1 obesity were included, of which 42 patients completed the intervention (52%); we defined as conclusive those patients who had all the evaluations, from baseline (visit 1) to visit 6. Of the patients who completed the intervention, the distribution by sex was 52% (n=22) females and 48% (n=20) males, of whom 64% (n=27) were school children and 36% (n=15) adolescents. As for the mean age, it was 10.91 ± 1.96 years for the freely chosen exercise (n=20) and 11.18 ± 2.03 years for the video-based exercise program (n=22), there was no significant difference between the mean age of both groups (p = 0.97) (Table 6).

**Table 6:** Baseline demographic, anthropometric, and metabolic characteristics of the study groups FCE: Free-choice exercise; EPV: Exercise program with videos; BMI: Body mass index; HDL: High density lipoprotein; LDL: Low density lipoprotein; HOMA-IR: Insulin resistance homeostasis model assessment. Children: < 12 years. Adolescents: ≥ 12 years. Student’s t-test was used for normally distributed variables, data shown as mean (± SD); Mann-Whitney U test for non-normally distributed variables*, data shown as median (IQR), and Chi-square tests for categorical variables†. A p-value < 0.05 was considered statistically significant.

Variable	FCE (n=20)	EPV (n=22)	p-value	Test statistics	
Sex (Boys: Girls)	7:13	13:9	0.118^†^	2.438^†^	
Age group (Children: Adolescents)	13:7	14:8	0.927^†^	0.008^†^	
Tanner scale			0.789^†^	1.710^†^	
1	7	9			
2	6	5			
3	1	1			
4	4	6			
5	2	1			
Age (year)	10.91 (± 1.96)	11.18 (± 2.03)	0.979	-0.384	
Systolic blood pressure (mmHg)	103.64 (± 8.20)	108.14(± 12.04)	0.191	0.191	
Diastolic blood pressure (mmHg)	67.10 (± 7.21)	69.59 (± 7.63)	0.201	-1.084	
Anthropometric variables					
Waist Circumference (cm)	84.88 (± 7.42)	86.97 (± 7.82)	0.953	0.953	
Weight (kg)	57.60 (± 11.65)	62.17 (± 12.18)	0.899	0.889	
Height (cm)	146.49 (± 8.14)	149.84 (± 10.12)	0.455	0.455	
BMI Percentile	97.42 (± 1.06)	97.61 (± 1.28)	0.212	0.212	
Body fat mass (kg)	18.28 (± 6.49)	19.44 (± 5.84)	0.596	-0.313	
Lean body mass (kg)	38.72 (± 5.81)	41.85 (± 7.00)	0.647	0.565	
Biochemical variables					
HOMA-IR	4.35 (± 2.6)	4.57 (± 2.18)	0.323	0.323	
Glucose (mg/dl)	90.65 (± 7.94)	90.00 (± 5.68)	0.119	0.302	
Insulin (µIU/ml)	19.05 (± 10.58)	20.57 (± 9.63)	0.455	-0.489	
HDL cholesterol (mg/dl)	39.30 (± 5.98)	37.09 (± 7.44)	0.060	0.60	
LDL cholesterol (mg/dl)	99.10 (± 22.88)	104.45 (± 20.44)	0.764	0.764	
Total cholesterol (mg/dl)	155.10 (± 26.73)	158.18 (± 24.73)	0.899	-0.388	
Triglycerides (mg/dl)	147.50 (± 71.32)	136.18 (± 76.33)	0.813	0.494	
Alanine aminotransferase (U/L)	21.5 (29)	22 (29)	0.622*	-2.802*	
Aspartate aminotransferase (U/L)	26 (44)	25 (31)	0.596*	-2.929*	
Gamma-glutamyl transferase (U/L)	17 (37)	15.5 (18)	0.640*	-0.978*	
Uric acid (mg/dl)	5.37 (± 0.94)	5.77 (± 1.03)	0.942	-1.243	

Concerning the percentages of adherence to food by percentage of adequacy (p=0.84) and adherence to exercise by percentage of frequency and time of execution (p=0.052), no significant differences were observed between the two groups for the EPV program and the FCE. Although more boys were recruited in the FCE arm, there was no significant statistical difference between groups according to a Pearson Chi-square test. In Table 6, we detail both intervention groups' demographic, anthropometric, and metabolic characteristics at the beginning of the program, where we can see no significant differences between groups.

Anthropometric and body composition results

**Figure 2:**
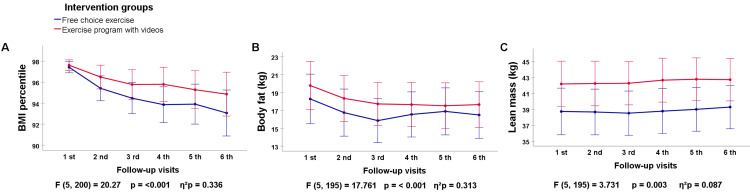
Differences in anthropometric variables between the FCE and EPV groups by one-way repeated measures ANOVA (visits 1-6) *p*: p-value between groups (ANOVA). Error bars indicate 95% confidence intervals. A p-value < 0.05 was considered as statistically significant. FCE: Free-choice exercise; EPV: Video-based exercise prescription program.

All variables were first evaluated using a one-way repeated measures ANOVA to assess differences between and within groups across visits 1 to 6. Student’s t-tests were conducted to compare initial and final measures within each group. The exercise intervention led to a significant decrease in BMI over time. The FCE group’s BMI percentile dropped from 97.42 ± 1.06 to 93.06 ± 5.87, while the EPV group’s BMI percentile declined from 97.61 ± 1.28 to 94.86 ± 3.66. Intragroup differences were significant in both the FCE (p = 0.002) and EPV (p < 0.001) groups (Figure 2).

Body fat mass also significantly decreased in both intervention groups. The FCE group reduced from 18.28 ± 6.49 kg to 16.49 ± 6.63 kg, while the EPV group dropped from 19.44 ± 5.84 kg to 17.65 ± 4.85 kg. Post hoc tests confirmed significant differences at all follow-up visits, and t-tests showed significant intragroup reductions (p = 0.001 for both groups) (Figure 2).

Lean body mass increased over time in both groups. The FCE group’s lean mass increased from 38.72 ± 5.81 kg to 39.27 ± 5.59 kg, and the EPV group from 41.85 ± 7.00 kg to 42.70 ± 6.37 kg. However, intragroup differences were not statistically significant (FCE: p = 0.12, EPV: p = 0.86), though lean mass was preserved throughout the intervention. Subgroup analyses by gender showed no significant differences (Figure 2). The differences in anthropometric variables between the FCE and EVP groups between visits 1 through 6 by one-way repeated measures ANOVA are shown in Table 7.

**Table 7:** Anthropometric parameters Differences in anthropometric parameters between the FCE and EVP groups between visits 1 through 6 by one-way repeated measures ANOVA*, and differences within groups (initial and final visits) by related-samples Student’s t test†. F: F statistic; p: p value; η²p: partial eta squared; FCE: Free-choice exercise; EPV: Video-based exercise prescription program.

Variable	Differences in anthropometric parameters between the FCE and EVP groups (visits 1 through 6)*			Interaction between Group and Time	Initial vs final^†^	Initial vs final^†^	Effect direction
					FCE group	EPV group	
	F	p	η²p	p	p	p	
BMI percentile	20.27	0.001	0.336	0.290	0.002	<0.001	Decrease (Both groups)
Body fat mass	17.761	0.001	0.313	0.281	0.001	0.001	Decrease (Both groups)
Lean body mass	3.731	0.003	0.087	0.713	0.12	0.86	Increase (Both groups)

All variables were also evaluated using a one-way repeated measures ANCOVA to assess differences between and within groups from visits 2 to 6, controlling for the effect of visit 1. For BMI, no significant between-group differences were found. However, the within-group analysis showed a significant BMI reduction in both the FCE and EPV groups between visit 2 and visit 6 (p = 0.002), transitioning participants from the obese category to the overweight range. For body fat mass, no significant between-group differences were detected. However, the within-group analysis showed a significant decrease between visit 2 and visit 3 in the FCE group (p = 0.042) after adjusting for baseline body fat mass. Concerning lean body mass, no significant between-group differences were found. However, after controlling for baseline lean body mass, within-group analysis showed a significant increase in the FCE and EPV groups between visit 2 and visit 6 (p = 0.028). These findings indicate that while both interventions had a similar impact on body composition, significant within-group improvements were observed for BMI, body fat mass (FCE group), and lean body mass over time (S1 Figure, Appendices).

A one-way repeated measures ANOVA showed a significant increase in height over time in both intervention groups (p < 0.001, η²p = 0.618). The FCE group increased from 146.49 ± 8.14 cm to 149.27 ± 7.41 cm, while the EPV group grew from 149.84 ± 10.12 cm to 153.57 ± 9.54 cm. The interaction between time and group was not significant (p = 0.292). There were significant differences between most time points (p < 0.001), except between visit 4 and visit 5 (p = 0.140) (S2 Figure, Appendices). Additionally, the growth velocity of the participants during the intervention was assessed. Prepubertal patients (Tanner 1) had an average growth rate of 8.3 cm per year, and early pubertal patients (Tanner 2-3) had an average growth rate of 9.1 and 6.5 cm per year, respectively. Late pubertal patients (Tanner 4) had an average growth rate of 4.3 cm per year, and patients in the final growth phase (Tanner 5) had an average growth rate of 1.8 cm per year. No significant differences were observed between intervention groups, confirming normal growth patterns during the study.

Metabolic results

**Figure 3:**
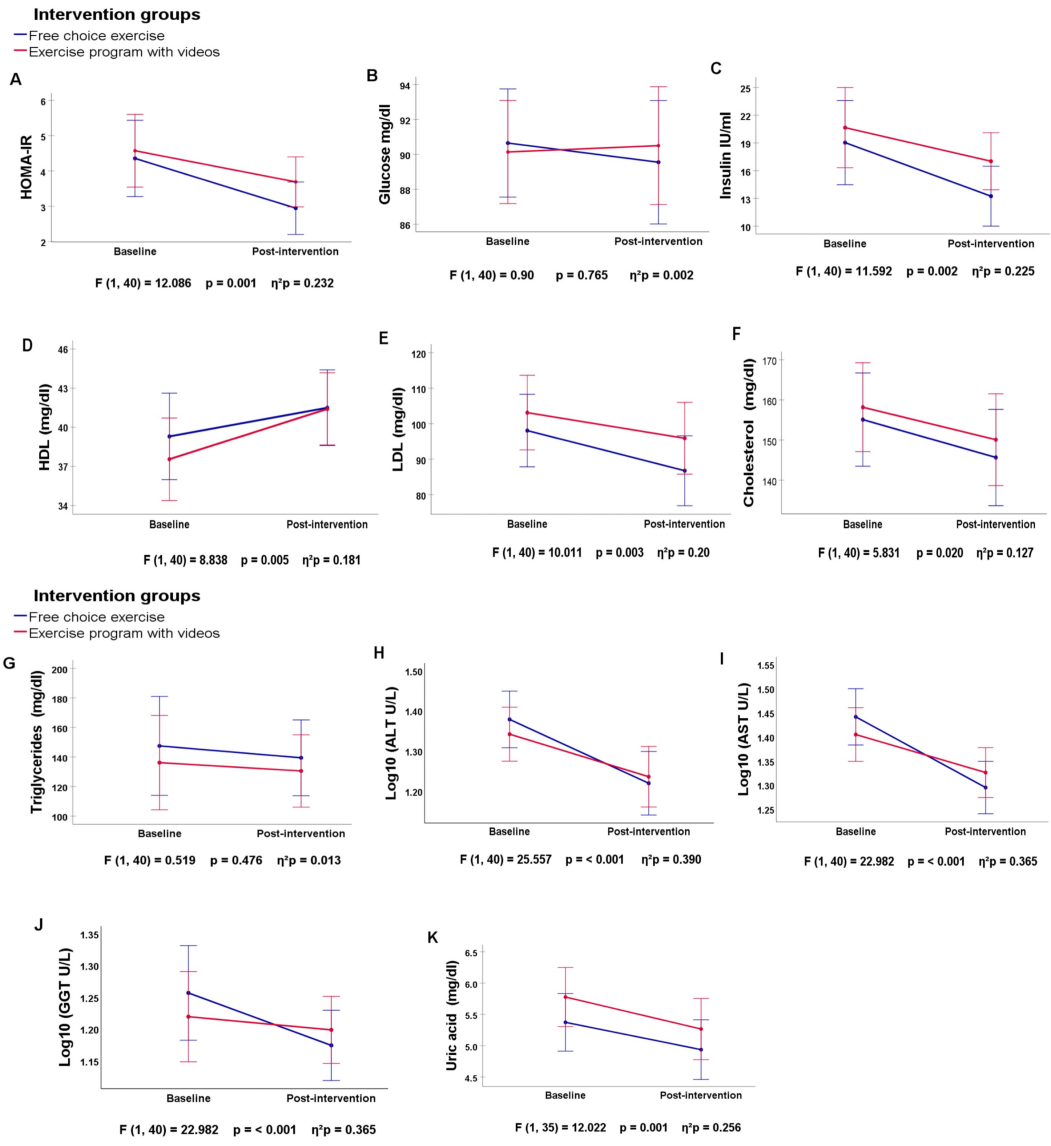
Differences in biochemical parameters between the FCE and EVP groups (visits 1 and 6), by one-way repeated measures ANOVA FCE: Free-choice exercise; EPV: Video-based exercise prescription program; p: p value, between groups. Error bars indicate 95% confidence intervals. A p-value < 0.05 was considered as statistically significant.

All variables were first evaluated using a one-way repeated measures ANOVA to assess differences between and within groups from visits 1 and 6 (Figure 3). The intervention led to a significant improvement in HOMA-IR over time (p = 0.001), but the interaction between time and group was not significant (p = 0.428).

Student’s t-tests showed a significant decrease in HOMA-IR in the FCE group (p = 0.006), while no significant change was observed in the EPV group (p = 0.07). Glucose levels remained stable, with no significant changes over time (p = 0.765) or within groups (FCE: p = 0.53, EPV: p = 0.83). On the other hand, insulin levels improved in both groups across visits (p = 0.002), but only the FCE group showed a significant reduction (p = 0.006). Concerning lipid profiles, HDL significantly increased (p = 0.005), with a notable improvement in the EPV group (p = 0.002) but no significant change in the FCE group (p = 0.60). LDL significantly decreased over time (p = 0.003), with reductions in both the FCE (p = 0.02) and EPV groups (p = 0.04). Total cholesterol also decreased over time (p = 0.020), but within-group comparisons showed no significant differences (FCE: p = 0.12, EPV: p = 0.08). Triglycerides did not change significantly (p > 0.05). For hepatic metabolism variables, ALT, AST, and GGT were Log10 transformed due to non-normal distribution. ALT, AST, and GGT significantly decreased over time during the intervention (p < 0.001 to p = 0.009). Both groups showed a reduction in ALT (p = 0.002) and AST (FCE: p = 0.004, EPV: p = 0.001). However, GGT significantly decreased only in the FCE group (p = 0.025), with no changes in the EPV group (p = 0.261). Lastly, uric acid significantly decreased (p = 0.001), with a reduction in the EPV group (p = 0.003) but no change in the FCE group (p = 0.44) (Figure 3). Overall, these findings indicate that both interventions led to metabolic and lipid improvements, with HOMA-IR, insulin, and GGT decreasing more in the FCE group. In contrast, the EPV group showed greater improvements in HDL and uric acid levels. The differences in metabolic variables between the FCE and EVP groups between visits 1 through 6 by one-way repeated measures ANOVA are shown in Table 8.

**Table 8:** Biochemical parameters Differences in biochemical parameters between the FCE and EVP groups between visits 1 and 6 by one-way repeated measures ANOVA*, and differences within groups (initial and final visits) by related-samples Student’s t test†. HDL: High density lipoprotein; LDL: Low density lipoprotein; HOMA-IR: Insulin resistance homeostasis model assessment; ALT: Alanine aminotransferase; AST: Aspartate aminotransferase; GGT: Gamma-glutamyl transferase; F: F statistic; p: p-value; η²p: partial eta squared; FCE: Free-choice exercise; EPV: Video-based exercise prescription program.

Variable	Differences in biochemical parameters between the FCE and EVP groups (visits 1 and 6)*			Interaction between Group and Time	Initial vs final^†^	Initial vs final^†^	Effect direction
					FCE group	EPV group	
	F	p	η²p	p	p	p	
HOMA-IR	12.086	0.001	0.232	0.428	0.006	0.07	Decrease (Both groups)
Glucose	0.90	0.765	0.002	0.553	0.53	0.83	No change
Insulin	11.592	0.002	0.225	0.441	0.006	0.08	Decrease (FCE group)
HDL	8.838	0.005	0.181	0.968	0.60	0.002	Increase (EPV group)
LDL	10.011	0.003	0.20	0.995	0.02	0.04	Decrease (Both groups)
Total cholesterol	5.831	0.020	0.127	0.853	0.12	0.08	Decrease (No group-specific effect)
Triglycerides	0.519	0.476	0.013	0.900	0.61	0.62	No Change
ALT	25.557	0.001	0.390	0.318	0.002	0.002	Decrease (Both groups)
AST	22.982	0.001	0.365	0.157	0.004	0.001	Decrease (Both groups)
GGT	7.609	0.009	0.160	0.107	0.025	0.261	Decrease (FCE group)
Uric Acid	12.022	0.001	0.256	0.787	0.44	0.003	Decrease (EPV group)

Lastly, we conducted one-way ANCOVA tests to compare final HOMA-IR, glucose, insulin, HDL, LDL, cholesterol, ALT, AST, GGT, and uric acid between intervention groups, adjusting for initial conditions. No significant differences were found in any of the analyses (S3 Figure, Appendices).

## Discussion

The main finding of this clinical trial was that both the freely chosen exercise format and the video-based exercise program, as part of the multidisciplinary intervention in childhood obesity, had favorable effects on body composition and metabolic parameters, as reported by Seo et al. [33] and Seabra et al. [34]. In modifying the energy balance, the expenditure that exercise promotes by the energy demand at the hepatic and muscular level favors the mobilization of fatty acids from adipose tissue, evidenced by the decrease in body fat mass, as observed in our intervention groups [35,36].

One aspect to highlight in our study is that none of the intervention groups had a loss of muscle mass, unlike studies whose objective is weight loss through exercise and caloric restriction [37,38]. We consider that in our study, having accompanied the exercise prescription with the indication of a supervised food plan prevented the loss of muscle mass that could even worsen the metabolic conditions of the patients [39,40].

Children and adolescents living with obesity present with endocrine and musculoskeletal alterations that contribute to an altered growth pattern. In addition, a pronounced weight loss can affect pubertal development and, therefore, the growth speed. Thus, as part of the evaluation and follow-up of our study, the height and growth velocity of the participants were evaluated during the intervention, identifying that the growth velocity was adequately maintained in both intervention groups. Adequate treatment in children and adolescents living with obesity does not affect but instead normalizes the growth velocity pattern, as reported by Putri et al. [41].

At the metabolic level, exercise promotes glucose uptake by increasing its delivery, transport, and intracellular metabolism, improving insulin sensitivity [42]. In our case, the freely chosen exercise group, which overall had a mostly aerobic profile, decreased significantly in insulin and HOMA-IR levels, as has been reported in clinical trials [43] and in a systematic review by Marson et al. [44], in which he suggested that aerobic training produced a superior beneficial effect on insulin sensitivity compared to resistance or combined training modalities.

Childhood obesity is associated with increased triglycerides, low-density lipoprotein cholesterol, and decreased high-density lipoprotein cholesterol. The presence of dyslipidemia in this population has been associated with the risk of premature atherosclerosis [45]. The scientific evidence on the relationship between exercise and lipid profile levels in children and adolescents living with obesity is contradictory. In our case, our intervention groups had a decrease in LDL cholesterol, and there was a significant increase in HDL cholesterol only when the prescription of exercise was through videos, as reported in the systematic review and meta-analysis study by Tianhao Chen et al. [46]. The effects on lipid profiles depend on variables such as exercise intensity, frequency, and duration, as well as the time and methodology employed [47].

The study by Seo et al. did not observe an association between exercise and lipid profile modification in the multidisciplinary lifestyle intervention program aimed at children and adolescents with obesity lasting four months [33]. However, Seabra et al. [34] in an intervention with a duration of six months, observed improvement in triglycerides, LDL, total cholesterol, and an increase in HDL, suggesting that the time of intervention is essential to observe some benefit in the lipid profile, which is a strength that our study has.

The observed prevalence of metabolic dysfunction-associated steatotic liver disease (MASLD) in children with obesity is clinically relevant and a significant public health concern, as it increases the risk of liver disease, cardiovascular disease, and mortality [48]. Few studies have analyzed the association between exercise and the percentage of hepatic fat infiltration, GGT, AST, and ALT levels in children and adolescents with obesity. Medrano et al. [49] found that greater cardiorespiratory fitness is associated with a lower percentage of liver fat and a healthier liver enzyme profile in prepubertal children with overweight and/or obesity.

A systematic review and meta-analysis conducted by González-Ruiz et al. [50] shows that physical exercise is an effective intervention in the treatment of hepatic steatosis by reducing visceral adipose tissue, subcutaneous adipose tissue, and GGT in overweight and/or obese children and adolescents, observing that aerobic training and programs of more than three sessions per week showed a greater reduction in visceral adipose tissue and subcutaneous adipose tissue. This is consistent with the findings in our study, where we observed improvement in AST and ALT levels in both intervention groups and a decrease in GGT levels in the freely chosen predominantly aerobic exercise group.

An increase in serum uric acid levels in children and adolescents is associated with increased weight and blood pressure values that may contribute to cardiovascular risk factors with a negative prognostic implication on the risk of cardiometabolic disease and kidney injury in the future [45,51]. Regular exercise and decreased sedentary time are associated with reduced levels of asymptomatic obesity-mediated hyperuricemia [52]. However, data on the effect of lifestyle interventions on uric acid in children and adolescents living with obesity are very scarce, as it is not a variable that is consistently assessed in intervention studies.

Cordellat et al. [53], who conducted a pilot study in children and adolescents with obesity with duration of four months of multimodal intervention comprising supervised multi-component exercise, periodized together with professional nutritional counseling, observed no significant differences in serum uric acid levels, unlike Krzystek-Korpacka et al. [54] who demonstrated a beneficial effect of lifestyle modification through diet and exercise for one year in overweight and obese children and adolescents in decreasing serum uric acid levels. In this same sense, through videos, our study demonstrates a beneficial effect in the intervention group. However, further research is required on the variables such as frequency, intensity, time, and type of exercise that contribute to obtaining this beneficial effect in children and adolescents living with obesity.

Although our study has shown that the two exercise modalities (freely chosen exercise and exercise through videos) are effective as part of the multidisciplinary treatment for obesity in children and adolescents, long-term outcome data are required to assess whether the increase in exercise and the observed benefits of exercise are sustained in the long term. Another limitation of our study was the small sample size and the loss to follow-up rate, which, although significant, is common in the population studied because of patient compliance or external factors. Patients who dropped out of the study were contacted, and the reasons for non-attendance were mainly due to changes in home address and maternal or paternal difficulties in obtaining permission to attend follow-up sessions. Loss to follow-up was not related to the prescribed exercise intensity or frequency. It is also important to note that the project had to be suspended due to the COVID-19 pandemic when the study was conducted. Because several participants did not finish follow-up, a missing data analysis was carried out, observing that the data was missing completely at random.

Due to the trends shown, we consider it valuable to carry out this study in a larger number of individuals. In our study, we did not apply any psychological instrument concerning the measurement of the effects on mental health and long-term adherence to exercise, which is a limitation of our research since it is well-known that positive experiences tend to achieve a greater attachment and pleasure for exercise, improving the quality of life.

Among the strengths of the study, it is essential to mention that the project sought to build a multidisciplinary lifestyle intervention treatment proposal aimed at children and adolescents living with obesity, with a particular focus on exercise prescription based on the anthropological characteristics of the population attended at our clinic, and easily accessible through videos so that children and adolescents could execute them at home, since this format of exercise at home offers flexibility and privacy to participants, providing accessibility and convenience for families limited by work, financial, location or transportation commitments. Among the most noteworthy results of this study was the improvement in body composition and cardiovascular risk markers, particularly with the home exercise modality, which may have a particular application among families with low economic and social resources who also live in unsafe environments.

## Conclusions

According to the results, this simple video-based exercise program, as a complement to a multi-component intervention program for healthy lifestyle changes, can be effective, feasible, easily accessible and have an impact on the cardiometabolic health of children and adolescents, as well as freely chosen exercise, as long as both are appropriately prescribed. This study provides evidence that proper exercise prescription is essential to ensure the beneficial effects of active games or exercises on the health of children and adolescents living with obesity.

## Figures and Tables

**Figure 4:**
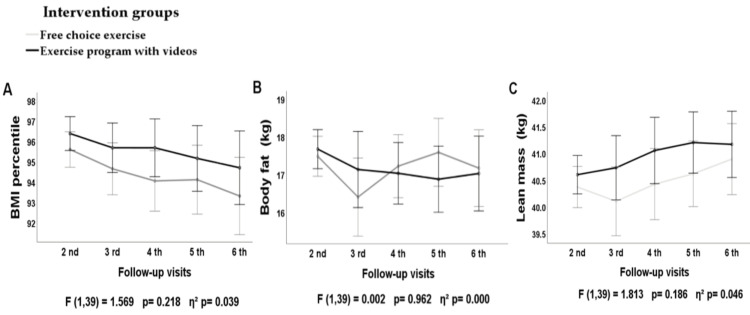
S1 Figure. Differences in anthropometric variables between the FCE and EPV groups by one-way repeated measures ANCOVA (visit 2-6), adjusting for basal anthropometric values (visit 1). p: p value between groups (ANCOVA). Error bars indicate 95% confidence intervals. A p-value < 0.05 was considered as statistical significance.

**Figure 5:**
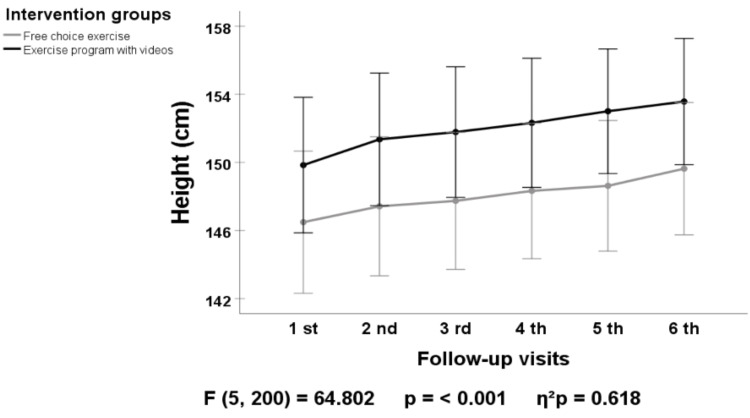
S2 Figure. Differences in anthropometric variable height between the FCE and EPV groups by one-way repeated measures ANOVA (visit 1-6). p: p value between groups (ANOVA). Error bars indicate 95% confidence intervals. A p-value < 0.05 was considered as statistically significant.

**Figure 6:**
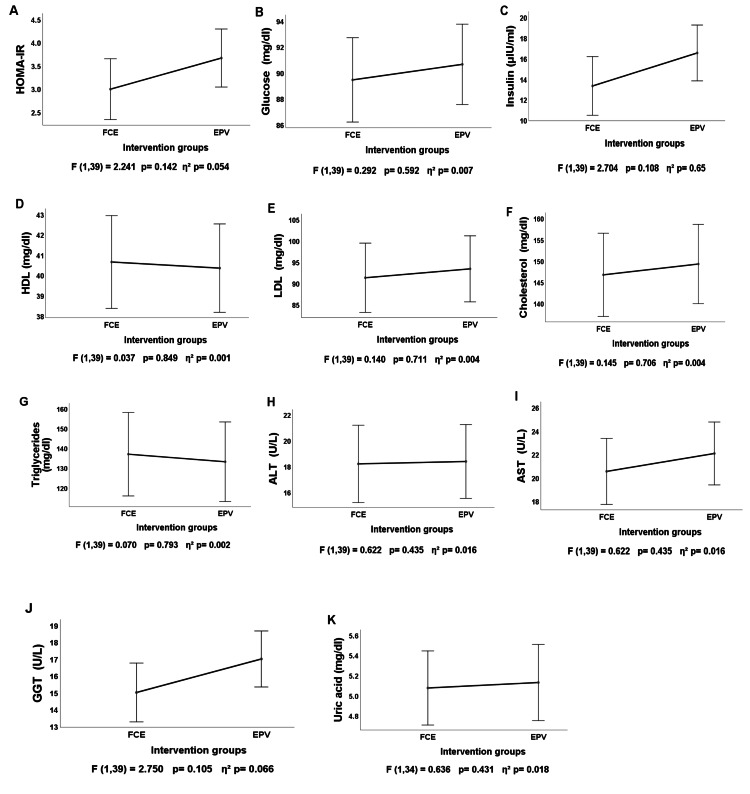
S3 Figure. Differences in final biochemical parameters between the FCE and EVP groups, by one-way ANCOVA, controlling for the initial biochemical parameters. p: p value, between groups. Error bars indicate 95% confidence intervals. A p-value < 0.05 was considered as statistical significance.
